# “When you talk it out … you will feel like the burden has somehow gone down, you will feel light”: Social Support Received by Gay, Bisexual, and Other Men Who Have Sex with Men in Western Kenya

**DOI:** 10.3390/ijerph19031667

**Published:** 2022-02-01

**Authors:** Laura Jadwin-Cakmak, Kendall Lauber, Elijah Ochieng Odhiambo, Ben Collins, Edwin Gumbe, Gabriella A. Norwitz, Teddy Aloo, Katherine A. Lewis, Felix Okutah, K Rivet Amico, Kennedy Olango, Wilson Odero, Susan M. Graham, Gary W. Harper

**Affiliations:** 1 Department of Health Behavior and Health Education, University of Michigan School of Public Health, Ann Arbor, MI 48109, USA; klauber@umich.edu (K.L.); bcol@umich.edu (B.C.); ramico@umich.edu (K.R.A.); gwharper@umich.edu (G.W.H.); 2 Nyanza Reproductive Health Society, Kisumu 40100, Kenya; almonelly@gmail.com (E.O.O.); gumbeedwin@gmail.com (E.G.); teddybrn@gmail.com (T.A.); felixokutah@gmail.com (F.O.); 3 Nafasi Innovations, Tom Mboya, Kisumu 40100, Kenya; 4 Department of Epidemiology, Mailman School of Public Health, Columbia University, New York, NY 10032, USA; gan2126@cumc.columbia.edu; 5 Department of Community Health Sciences, Fielding School of Public Health, University of California Los Angeles, Los Angeles, CA 90095, USA; katlew@ucla.edu; 6 Men Against AIDS Youth Group (MAAYGO), Kisumu 40100, Kenya; kenolango37@gmail.com; 7 School of Public Health and School of Medicine, Maseno University, Kisumu 40100, Kenya; wilsonodero@gmail.com; 8 Global Health, Medicine, and Epidemiology, School of Medicine, University of Washington, Seattle, WA 98104, USA; grahamsm@uw.edu

**Keywords:** Kenya, LGBTQ, social support, mental health, gay and bisexual men, men who have sex with men

## Abstract

Gay and bisexual men and other men who have sex with men (GBMSM) experience many sexual orientation-related stressors that negatively influence physical and mental health, making it imperative to understand their experiences of resilience-promoting resources such as social support. We utilized qualitative and participatory methodologies to examine sources of social support and types of social support received by GBMSM in Western Kenya through in-depth interviews with 60 GBMSM, including both peer educators and community members. GBMSM received emotional, informational, and instrumental support from six different relationship types: friends and peer groups, family of origin, sexual and romantic partners, healthcare providers, peer educators, and other people including work colleagues and police officers. A key finding from this study is the centrality of sexuality-specific support across all sources and types of support. Implications for clinics and LGBTQ organizations, policy, and future research are discussed.

## 1. Introduction

Currently, anti-lesbian, -gay, -bisexual, -transgender, and -queer (LGBTQ) structural and socio-political conditions in Kenya create a harsh environment for gay and bisexual men. In Kenya, same-sex behavior is punishable by up to 14 years in jail [1]. Though local LGBTQ human rights organizations petitioned the High Court of Kenya to declare the relevant sections of the penal code unconstitutional, thus decriminalizing same-sex behavior among consenting adults, in 2019 the High Court rejected this petition and upheld the criminalization [2,3]. This further emboldened some members of the public as well as religious leaders to harass LGBTQ Kenyans. Though same-sex behaviors and relationships have often been characterized by African religious and political leaders as “un-African” and a result of Western colonialization, scholars have noted substantial historical evidence of a variety of same-sex behaviors and relationships and diverse gender identities existing across the African continent before colonialization. In fact, the laws and policies prohibiting same-sex behaviors were only initiated on the continent during the period of colonization and have been maintained through to the present [2,4,5]. The combination of these legal and socio-cultural conditions creates an environment in which gay and bisexual men in Kenya commonly encounter stigma, discrimination, and violence across socio-ecological levels. A number of research studies have documented the human rights violations faced by gay and bisexual men and other men who have sex with men (GBMSM), including verbal harassment, physical violence, mob justice, and sexual violence, perpetrated by religious leaders and congregations, families, neighbors, police, and landlords, as well as within educational settings and workplaces [6,7,8,9,10]. GBMSM have also reported experiences of discrimination, harassment, and denial of care by healthcare providers, in addition to a lack of knowledgeable providers, leading some to delay or avoid needed care [11,12].

The Minority Stress Model provides a framework for understanding how prejudice and stigma directed toward LGBTQ people bring about unique stressors that cause adverse health outcomes [13,14]. These minority stress processes include those that are external, such as being discriminated against or being the target of violence because of one’s sexual orientation, as well as those that are internal, such as expectations of rejection, concealment of one’s identity, and internalized homophobia. Though initially developed and used with lesbian and gay populations in the United States, the minority stress model has since been widely used with LGBTQ populations internationally, including in the sub-Saharan African countries of Zambia, South Africa, and Nigeria [15,16,17,18,19,20]. Using a minority stress framework, given the pervasive stigma and violence that GBMSM experience, one would expect to see poor mental health outcomes among GBMSM. Research has indeed documented inequities in mental health outcomes among GBMSM in Kenya, including a high prevalence of clinically significant depressive symptoms [7,8,10,20] and post-traumatic stress disorder (PTSD) symptoms [20], as well as elevated rates of alcohol and other substance abuse among gay and bisexual men [7,8,10]. Additionally, in a qualitative study with gay and bisexual men in coastal Kenya, participants described experiences of stigma and discrimination as contributing to their mental health challenges or substance abuse [11].

Given the high prevalence of sexual orientation-related stressors experienced by GBMSM in Kenya and the implications of these minority stressors for health and well-being, it is important to have a thorough understanding of the adaptive coping strategies that help GBMSM survive and thrive [21]. Social support—one such resilience-promoting factor—refers to interpersonal interactions that are meant to be helpful. Social support is most often conceptualized as having three primary dimensions: emotional (i.e., expressions of empathy, love, trust, and caring), informational (i.e., advice, suggestions, and information), and instrumental (i.e., tangible aid and service) [22,23,24,25]. A large body of literature on studies among diverse populations shows the consistent association of social support with positive health outcomes, whether related to mental health, recovery from trauma, or chronic disease [26,27,28]. There is limited research focused on Kenyan GBMSM’s experiences of receiving social support. In a qualitative study of facilitators and barriers to antiretroviral therapy (ART) adherence among Kenyan GBMSM living with HIV, Graham et al. [11] found that many interviewees spoke of the importance of trusted healthcare providers and supportive family and friends, as well as their connection to the local LGBTQ community, in helping them successfully adhere to ART [11]. Several quantitative studies have also identified connections between social support and resilient health outcomes among GBMSM in Kenya. Harper and colleagues [29] found that higher perceived social support was correlated with sexual health outcomes including increased condom use and increased likelihood of HIV testing, as well as mental health outcomes including higher self-esteem and lower levels of both depression and anxiety; additionally, lower levels of loneliness were associated with lower levels of depression and anxiety, as well as a greater likelihood of intention to test for HIV in the next 3 months [29]. A recent study by Doshi and colleagues [30] with male sex workers (MSW) and other GBMSM in Nairobi found that those who were members of an organization that supported MSW and other GBMSM had greater quality of life, reported higher levels of social support, and were more likely to have ever accessed mental health services than non-members.

While the evidence is clear that social support can contribute to improved health and well-being, it is also clear that not all social support is equal. Research using quantitative measures of social support—for example, increases in social contact, social interaction, and the provision of social resources—finds that these aspects of social support are not always health-protective [27]. In fact, many of the characteristics of social environments and relationships that are presumed to be beneficial are not associated with better health, and attempts to improve health and well-being through planned social support interventions have had mixed success [27]. This makes it clear that not all types of social support improve an individual’s health. Beyond simply noting the provision (or perceived availability) of social support, a nuanced approach that takes into account additional considerations such as who provides the support and the quality of that social relationship, is warranted. This nuanced approach may be especially important when investigating the phenomenon of social support among populations that are stigmatized, such as Kenyan GBMSM.

The current study extends the literature on the different types of social support received by GBMSM in Kisumu, Kenya and the ways in which that support contributes to their sexual health, including PrEP use, as well as their mental health and general well-being. This qualitative analysis investigates two research questions. (1) From whom do GBMSM in Kenya receive social support? (2) What types of social support do GBMSM describe receiving from these sources?

## 2. Materials and Methods

### 2.1. Participants

We utilized a 2 (PrEP experience) × 2 (PrEP interest) stratified purposive sampling frame to recruit 40 HIV-negative GBMSM with varying levels of PrEP experience and interest for individual in-depth interviews (IDIs). We refer to these participants as Community Members. This resulted in 10 PrEP-experienced GBMSM currently taking PrEP, 10 PrEP-experienced GBMSM who had stopped taking PrEP, 11 PrEP-naïve GBMSM interested in taking PrEP, and 9 PrEP-naïve GBMSM who had no interest in taking PrEP. Additionally, we conducted 20 IDIs with GBMSM who were working as peer educators in HIV testing, prevention, and treatment programs in the Kisumu area. We refer to these participants as Peer Educators.

Community Member participants met the following inclusion criteria: assigned male sex at birth and currently identify as a man, aged 18–30 inclusive, resident of Kisumu, reported at least one act of anal or oral intercourse in the previous 6 months with another man, self-reported as not living with HIV, and willing and able to provide informed consent and participate in an IDI. Peer Educator participants had all of the same inclusion criteria except HIV status and age, and the additional criterion of currently working as a peer educator or in similar role in an HIV testing, prevention, or treatment program in the Kisumu area. Overall, we sought to recruit individuals who were perceived to be good key informants, defined as a person who thinks about the study topics, is comfortable talking about these topics, and is good at describing their thoughts and feelings.

Community Members ranged in age from 20 to 30 (mean = 26.4) and the majority identified as bisexual (47.5%), whereas Peer Educators ranged in age from 22 to 45 (mean = 26.6) and the majority identified as either gay (35%) or bisexual (also 35%). The majority of Community Members had attained a diploma (a two-to-three-year course completed post-secondary school) and were working part-time, while the majority of Peer Educators had completed secondary school and were working part-time. The majority of both groups were Christian and Luo, and the majority of Peer Educators had been working as such for 1 to 2 years (See Table 1).

### 2.2. Qualitative Interview Guide

The research team, which included researchers with extensive experience working with Kenyan GBMSM and local collaborators who were themselves GBMSM, created a semi-structured qualitative interview guide for the parent study. Throughout the course of qualitative interviewer training, modifications were made to the guide to ensure its utility with regard to GBMSM in Kisumu. Grounded in phenomenological and constructivist frameworks, the guide provided a general structure for discussion but required participants to provide their own conceptualizations of terms and phrases based on their lived experiences. The guide included a series of questions/probes focused on four primary areas based on the parent study’s research questions: health issues affecting GBMSM, thriving/coping as a GBMSM, experiences with PrEP, and recommendations for improving PrEP services for GBMSM. The structure and content of the questions did not follow any a prior theory or framework, which allowed us to conduct an inductive inquiry into participants’ thoughts, feelings, and experiences in these general areas.

### 2.3. Procedures

Given the purposive nature of our sampling frame, we recruited participants through outreach activities conducted by our interviewers at community-based organizations (CBOs) and health clinics in Kisumu who provide services to GBMSM. Our research team has worked with these CBOs and clinics for more than 10 years, and has conducted participant recruitment for other GBMSM-focused studies using similar procedures in these venues. Recruitment and screening took place verbally with GBMSM who fit the criteria, in accordance with our inclusion criteria and our stratified sampling framework. Interviews took place in private rooms at one of our CBO or clinic research sites. Interviewers obtained verbal consent for research participation, then verbally administered a brief demographic survey and conducted the IDI, which was audio-recorded. The interviewer debriefed with the participant after the interview, provided him with a monetary incentive, and shared information about local GBMSM-friendly resources and services. The interviewer then completed a written post-interview summary with details about his overall impressions of the interview, key information provided, PrEP-specific information, and participant recommendations. Interviews were conducted in a mix of English, Dholuo, and/or Kiswahili, based on the most comfortable language for the participant. A local transcriptionist experienced with GBMSM-focused research simultaneously translated and transcribed the recordings. Transcripts were de-identified and quality-checked to ensure accuracy of transcription. The Institutional Review Boards of the University of Washington and the University of Michigan, as well as the Maseno University Ethics Review Committee, provided approval for, and oversight of, the research protocol.

### 2.4. Data Credibility and Analysis

We used several strategies to enhance credibility during data collection and to increase alignment during data analysis between the perspectives shared by participants and our representation of those perspectives: prolonged engagement, persistent observation, triangulation, and member checking [31,32]. With regard to prolonged engagement and persistent observation, senior members of our research team have worked with the broader LGBTQ community in Kisumu for more than 10 years, with a primary focus on GBMSM. These interactions have not only included research activities, but also capacity-sharing and program development activities, social and cultural events, and community organizing. In addition, some members have provided medical and mental healthcare and services to GBMSM in the community through local community clinics and CBOs. Through these in-depth and prolonged interactions and activities, the research team has gained a high degree of trust from members of the community. We also implemented various types of triangulation methods by using five different interviewers to collect data, collecting data from two different groups of key informants (Community Members and Peer Educators), and working with six different analysts of varying educational levels and disciplinary backgrounds (public health and psychology). Finally, we engaged in member checking by presenting the results to four of the five interviewers (all GBMSM from Kisumu with extensive work and/or advocacy experience within the local LGBTQ community) and having them provide feedback and confirmation regarding the analytic findings.

The current analysis sought to explore the various ways in which GBMSM in Kenya benefit from social support despite pervasive stigma and discrimination against sexual minorities throughout the country. Since the focus of this study is on the lived experiences of GBMSM in Kenya, we conducted the analyses using a phenomenological inquiry framework [31,32,33]. Phenomenology is specifically focused on describing what a given group of people have in common as they experience the same or similar experiences or phenomena and is an inductive analytic approach that allows the patterns, themes, and categories of the analysis to emerge from the voices of participants. The composite descriptions of the phenomena of social support presented in this article explain the underlying structure that exists across participants [32,34]. In line with our phenomenological framework, we conducted the analyses to ensure the representation of different voices in the findings and to ensure that dominant perspectives did not silence conceptual “outliers”. Thus, we present all voiced themes instead of only those endorsed by a majority of participants [32,34].

The analyses were conducted by a group of six analysts from the U.S., representing various educational levels: undergraduate students, graduate students, a Master’s-level staff member, and a doctoral-level faculty member. Both the staff member and the faculty member have extensive experience working in Kenya, are primary investigators on the parent study, and trained and supervised the interviewers in Kenya. Student analysts were given required background reading to familiarize them with both the study population and the qualitative analysis. In addition, analysts read all 60 of the post-interview summaries provided by the interviewers, in order to gain an understanding of the nature and overall findings of the interviews.

We used an inductive consensus-building process to determine the focus of the current analysis. In addition to the 60 post-interview summaries, each member read five transcripts to brainstorm questions and common themes in marginal notes, with each person overlapping on only one transcript with another analyst. The team met on a weekly basis to discuss the content of the transcripts, and to build consensus on an area of focus for the analysis. Once a broad research question was developed (What does social support from different types of individuals look like for GBMSM in Kisumu, Kenya?), subsequent meetings involved reading more transcripts, reviewing notes to recognize key themes, identifying key sources and types of social support, and developing a formal codebook to name and operationally define each code.

All transcripts were then divided evenly between team members ensuring variability and overlap. Team members then participated in open coding, which involved applying codes to their assigned transcripts and noting key representative quotes. At least two different analysts reviewed each transcript in order to increase analytic dependability. As coding progressed, the team met weekly to review the codes and make modifications to the codebook, which involved eliminating, collapsing, or splitting codes. Discrepancies in coding were resolved through discussion and consensus-building.

## 3. Results

We identified six types of relationships from which GBMSM received various types of social support: friends and peer groups, family of origin, sexual and romantic partners, healthcare providers, peer educators, and other people including work colleagues and police officers. Three types of social support—emotional support, informational support, and instrumental support—were reflected in the participant discourse across these groups, though the ways in which each type of support was expressed varied by group (Table 2). Detailed descriptions of the emotional, informational, and instrumental support GBMSM received within each relationship type are provided with supporting quotes, along with a participant pseudonym and relevant demographic information (age, sexual orientation identity, and whether the participant was a Peer Educator or a Community Member).

### 3.1. Friends and Peer Groups

Participants described receiving emotional, informational, and instrumental support from friends and peer groups, defined as people with whom one has a mutual affectionate bond and who are not considered family members or romantic/sexual partners. Participants predominately described receiving social support from friends who were also GBMSM.

#### 3.1.1. Emotional Support from Friends and Peer Groups

Friends and peer groups provided emotional support to GBMSM by showing empathy and compassion. This support was most helpful in instances of uncertainty, hardship, or discrimination regarding sexual orientation and during mental health challenges. Participants discussed the importance of friendships with other GBMSM in helping them to know they are not alone and to cope with distress, even preventing suicide. Going out and having fun with friends was another source of emotional support.

“…when one is alone and is asking himself so many questions, why, why, why? They will never get answers, in return it is much dangerous because if you can’t get the answers, the next thing is to end your life or do something worse to yourself because if it cannot change then I will change the situation, some can change the situation by disappearing completely. So I will urge MSMs that whenever they feel low, they should engage. They should be together with other MSMs. like look for your friends, meet, have stories, talk, you make fun, you will forget your stress, that is when you will believe.”

Wickliffe, 20, Gay, Community Member

“Yes, because I have friends who are like me, so they have helped me a lot. When I see a friend who is like me, I say that yes, so I am not alone, I have—there are so many people like me.”

Harry, 29, Gay, Peer Educator

#### 3.1.2. Informational Support from Friends and Peer Groups

Friends and peer groups offered knowledge, facts, and advice related to health, well-being, and GMSM-friendly organizations. When asked about how GBMSM maintain their health, participants described a network of friends and peers who shared information on health topics and available GBMSM-affirming resources and services:

“Mmh mostly it is just… forums that are organized and people are called and sensitization is done or information is given, so you find that…there is something like a network, friends call friends, or if I know something that is going to happen somewhere I call someone and when you go there you get information or at least everyone has information.”

Nicholas, 27, Bisexual, Peer Educator

“…And MSMs are also good advisers to each other, especially those who have gone through the same situation, they will tend to make you be—to make you understand how they came out of it and in such a way the person who is the victim will get points and also information on how to cope with the ideas. So I would say looking for network, your connections I will say—your social connections will also help a lot.”

Wickliffe, 20, Gay, Community Member

#### 3.1.3. Instrumental Support from Friends and Peer Groups

Participants discussed ways that their friends and peers had helped them to become a part of the GBMSM community and to join affirming organizations, promoting a healthy sexual orientation identity. Friends and peer groups also helped other GBMSM learn how to cope with discrimination and victimization, and helped one another engage in health-promoting behaviors, e.g., by acting as “treatment buddies” and providing medication reminders for those on PrEP or ART.

“(I: How did you get involved with the LGBTQI community?) Ok…first I had a friend, who introduced me. After that he took me to some organization, where I could get support in terms of health issues. From there, that is how I got to know the organization’s program.”

Duncan, 26, Bisexual, Community Member

“Buddy, I think it is called treatment buddy or something of that measure, where you have your number and you set a certain time with your friend, peers and all that, so that when it reaches a certain time you can remind someone with maybe a text message, and say ‘you know it’s time you popped your pill?’ And you can send this message to many of your friends, so if somebody was to forget you can remind them that they are supposed to be taking it.”

Feshal, 30, Gay, Community Member

### 3.2. Family of Origin

Participants also described emotional, informational, and instrumental social support from families of origin. Though many participants did not identify their family of origin as a source of support, others with more GBMSM-affirming families did explain that this support was present and impactful in their lives.

#### 3.2.1. Emotional Support from Family of Origin

Families of origin provided emotional support by showing acceptance, compassion, and love, often in the form of affirming the participants’ sexual orientation, which helped them cope with stressors. Some participants described how family members accepted their sexual orientation, and the large positive impact of acceptance from one’s family of origin, while other spoke of receiving emotional support from family members without clarifying whether or not this was related to their sexual orientation, or whether or not family members were aware of their sexual orientation.

“I came out to my family members who are the people I treasure most in my life; they are the people I value most in my life. So once they accepted me being gay that has been my greatest support system…”

Newton, 28, Gay, Community Member

“(I: Now what do you think has helped other GBMSM to cope?) I think is how they carry out themselves, maybe if there are some who are being supported by the family—now if your family know that you are gay or transgender, [if] the support of the family is very strong, it can make you cope or fight any situation that comes your way.”

Kevin, 24, Transgender, Peer Educator

#### 3.2.2. Informational Support from Family of Origin

Family members provided informational support by offering knowledge, facts, and advice related to maintaining good health. Although family of origin was not discussed as frequently as a source of health information as other groups (e.g., friends, partners, healthcare providers), several participants shared that family members, including parents and siblings, provided them with sexual health prevention information and strategies.

“I protect myself, that is one, I use condoms, I go for checkups, I’m enrolled on PrEP that’s another and I get advised too… by friends, yeah, and the clinicians. (I: Who else?) Yeah, my father is one of them, yeah.”

John, 25, Bisexual, Peer Educator

#### 3.2.3. Instrumental Support from Family of Origin

Families of origin provided tangible support, which often involved financial support such as helping their child to get an education, as well as assisting with medical needs. Benson (23, Bisexual, Community Member) expressed gratitude for the financial support from his family that facilitated his education and thus helped him to learn about his legal rights as a sexual minority person: “I thank my folks for taking me to school, so that then means I know some of my rights. I might not know all because I am not a lawyer but then I know the basics that protect me.” When asked whether he knew of GBMSM who are on PrEP and supported by their family, Salim (28, Gay, Peer Educator) shared, “Yes I’ve seen so many, and they are urged to- to keep on taking dr–even the family reminds you of the time you are supposed to take the drugs [PrEP].”

### 3.3. Sexual and Romantic Partners

Sexual and romantic partners—those with whom participants had a sexual and/or romantic connection, including committed partners, casual partners, and hookups—also provided emotional, informational, and instrumental support.

#### 3.3.1. Emotional Support from Sexual and Romantic Partners

Sexual and romantic partners provided acceptance and understanding, as well as love and care, which served as a protective factor against outside discrimination. Emotional support from partners also included having open, honest discussions about sexual health and supporting health-related decisions.

“What has helped me survive as a bisexual? Ah that is I would say my partner, ah we understand the discrimination around it, and ah most people know us as friends not as my partner, so I will say it isn’t the best way to live but that is what has helped me cope.”

Joshua, 29, Bisexual, Community Member

“My partner. Aah, we first discussed with my partner to a certain point that now because I am with you and you alone, I feel I want to stop using PrEP as a preventive measure but I will focus on using condom. Then he didn’t disallow me, he also said yes because it was my decision.”

Dennis, 21, Gay, Community Member

#### 3.3.2. Informational Support from Sexual and Romantic Partners

When discussing social support from sexual and romantic partners, participants most often described emotional and instrumental support, although informational support was occasionally mentioned in the context of sexual health protection and decision-making. When asked about factors that have helped him protect his sexual health, Ian (23, MSM, Community Member) shared, “I get ideas from my friends and partner.” Another participant described:

“I think they [partners] ah, they used to encourage me, they used to take me for health sessions or health sensitization about PrEP, ah they also tell me the significance of using PrEP and the disadvantage of stopping to use it. These are my close friends, close GBMSM friends or how can I say it? Partners.”

Emmanuel, 24, Bisexual, Peer Educator

#### 3.3.3. Instrumental Support from Sexual and Romantic Partners

Sexual and romantic partners provided tangible support in the form of financial assistance, introducing their partners to LGBTQ organizations and GBMSM-friendly clinics and supporting their health by utilizing sexual risk reduction practices and providing medication reminders. Having partners who could provide financial support helped some GBMSM navigate socio-economic vulnerability. Another participant described how he was used to experiencing discrimination in standard hospital settings until his partner introduced him to a clinic that was safe and welcoming.

“Yes, I have a partner who has helped me to- maybe they have supported me financially, support my house rent, my upkeep, so it has really helped a lot to cope as MSM, so I don’t get financial difficulty because I have MSMs who are supporting me to pay my rent and buy food, stuff, yeah.”

Richard, 29, MSM, Peer Educator

“… now that is the partner talking, ‘I will take you somewhere, where you will be very free, even if you say you are gay there is no problem’ … I was like hospitals are nowadays so enlightened, let us go, so when we went the person took me to a LGBT organization and there I really received- I would say I received ah wonderful health service.”

Wickliffe, 20, Gay, Community Member

### 3.4. Healthcare Providers

Healthcare providers are defined here as people who are qualified to provide healthcare to community members in a professional setting, including clinicians and counselors who provide mental health support, many of whom are trained as HIV Test Counselors.

#### 3.4.1. Emotional Support from Healthcare Providers

Healthcare providers offer emotional support by treating their patients with respect and dignity and listening carefully to their patients’ needs, which contributes to an overall GMSM-affirming healthcare setting. While many participants described how some hospitals could be discriminatory toward GBMSM, they also explained how important it has been for them to interact with healthcare providers who accept them and listen to their needs.

“Okay at times it is good because when you talk it out with maybe clinicians or the other people around, you will feel like the burden has somehow gone down, you will feel light.”

Kevin, 24, Transgender, Peer Educator

“… being treated as a human being by the health service provider [helps me to feel sexually healthy].”

Amos, 30, Bisexual, Community Member

#### 3.4.2. Informational Support from Healthcare Providers

By providing credible and relevant informational support, healthcare providers were able to assist participants in making educated decisions about protecting their health. Healthcare providers shared credible and relevant information and assisted participants in making educated decisions about their health. They advised patients on sexual-health promotion tools such as condoms, lubricants, PEP, and PrEP.

“The clinician who enrolled me [on PrEP] encouraged me more than the friends. He explained it deeply, he convinced me more and I found it interesting.”

Peter, 25, Bisexual, Community Member

“Another thing is the advices I got from the doctor. You know after testing negative a doctor, a wise doctor will try to advise you to enroll on PrEP, so I was talked to by my health facility specialist on the benefits of being enrolled on PrEP of which I took them positively since I saw the benefits.”

Wycliffe, 20, Gay, Community Member

#### 3.4.3. Instrumental Support from Healthcare Providers

Healthcare providers offered instrumental support in the form of STI and HIV prevention and treatment, physical examinations and other routine medical procedures, medication prescription, and outreach to patients that may need check-ins or medication reminders outside scheduled clinic appointments. Instrumental support from healthcare providers included providing free or affordable services and reaching out to patients outside the clinic to retain even the most difficult-to-reach patients.

“It is good because ah the clinicians are even going out of their way to reach the clients with the PrEP wherever the clients are. There are some clients who don’t want to be seen in the organizations so through peer educators the clinician will reach that client at his or her comfort, and then the PrEP will be provided.”

Kevin, 24, Transgender, Peer Educator

“I also have a clinician who also reminds me, she will call every month even when I am not in town.”

Peter, 25, Bisexual, Community Member

### 3.5. Peer Educators

Peer educators are individuals who are GBMSM and who work or volunteer with a clinic or organization to assist and support the GBMSM community. Peer educators are accessible frontline workers who interface between clinics/organizations and community members, focusing on those who are most vulnerable due to poverty, health status, and/or victimization.

#### 3.5.1. Emotional Support from Peer Educators

As explained by the Community Members, peer educators affirmed community members’ sexual orientation identity, empathized with their experiences, listened to needs, and provided encouragement. Many participants expressed how having a close relationship with a peer educator helped them to accept themselves and cope with difficult life experiences:

“There are people in terms of where they work actually, the kind of activities they do, that is, they capacity build, they do orientation, and they create awareness. These are the people that have made me be aware that I exist, and you need to believe in your existence.”

Duncan, 26, Bisexual, Community Member

“He used to encourage us and tell us more about how we could cope with the community… During that time we used to use advice from the peer educators and that has played a major role in me.”

Augustine, 24, Bisexual, Community Member

#### 3.5.2. Informational Support from Peer Educators

As explained by the Community Members, peer educators provided informational support by providing knowledge, facts, and advice, especially related to sexual health and mental health. Many participants explained how peer educators had helped them in times of need by providing necessary information and advice:

“Now as people have been informed, people have been trained to teach others, and things like that help us so much. So in case you have a problem, you know if you go to such a person, he’ll help you handle it… In case you have health concerns, either you have STI, or things like that, I know where to go.”

Martin, 30, Bisexual, Community Member

“A second thing, we have peer education meetings and outreaches. Here you find different kinds of people with different mindsets. We share ideas, and also here you will be able to know each and every impact to each and every individual in the LGBTI community, and from there they can create awareness to areas where they are heading to or areas where they are situated in.”

Bernard, 28, Bisexual, Community Member

#### 3.5.3. Instrumental Support from Peer Educators

As explained by the Community Members, peer educators provide GBMSM with tangible support by taking community members to a GBMSM-affirming clinic, introducing them to people who can help them such as peers and leaders at LGBTQ organizations, and giving them condoms and lubricants. Participants explained how peer educators would go out of their way to help by scheduling appointments, connecting them with community activities, delivering medication, and providing transportation to the clinic:

“The peer educator whom I am linked to can always or sometimes do call me when I have… in case I need anything or if there is any activity within the project, the person always calls me or book appointments for me.”

David, 26, Gay, Community Member

“At times I might be in need of services and my peer educator can bring them [medication] to me, at times I might be sick and can come to the facility and get treated for free, they even come for me at home when I am sick.”

Joseph, 22, MSM, Peer Educator

### 3.6. Other People

While participants most often referred to receiving social support from the interpersonal relationships listed above, participants occasionally noted other types of people outside these groups as providing some types of support to GBMSM community members. Specifically, participants talked about emotional support from work colleagues and instrumental support from police officers.

#### 3.6.1. Emotional Support from Work Colleagues

Work colleagues were occasionally cited as a source of empathy and compassion, which included continuing to treat GBMSM with respect upon learning of their sexual orientation and supporting their health goals. Some participants identified work colleagues as an important source of support due to their welcoming attitudes, and connected this to their health:

“(I: What influences how you feel physically?) Let us say being in a community that you are free, being with colleagues that understand you, that one affects your health.”

Brian, 27, MSM, Community Member

Similarly, Victor (29, Gay, Peer Educator) explained how the support he received from his work colleagues made him feel more comfortable disclosing his gay sexual orientation identity (which he colloquially refers to as a “change of sexual orientation”): “I found my colleagues, some of them that I came to realize that they are loyal, very motivating, that is when I decided to change [disclose] my sexual orientation.”

#### 3.6.2. Instrumental Support from Police

Police officers were occasionally cited as a source of tangible support that helped to prevent and deal with human rights abuses to which community members were subjected. Although local police forces have often perpetuated discrimination against members of the GBMSM community, historically and in the present, a few participants explained how some police officers protected members of the LGBTQ community from violence and discrimination. One participant explicitly links this to ongoing local efforts to sensitize (described by the participant as “empowering”) police officers regarding LGBTQ human rights:

“Some policemen who understand, there are some policemen who understand after being empowered … and they are able to help any LGBTI persons in difficulties, yeah, understand their needs and everything that they need.”

Brian, 27, MSM, Community Member

“Right now, for example, there are officers that can help you when, for example, you feel like someone has discriminated or abused you, you can go and report. Sometimes I feel secured because of that.”

Joseph, 22, MSM, Peer Educator

## 4. Discussion

We identified six types of relationships from which GBMSM received various types of social support. The six sources of social support that participants discussed were friends and peer groups, biological family, sexual and romantic partners, healthcare providers, peer educators, and other people including work colleagues and police officers. Participants described receiving emotional, informational, and instrumental support across these groups; we identified both similarities and distinctions among the ways each type of social support—emotional, informational, and instrumental support—was expressed by each group. Social support is known to have positive impacts on mental health and sexual health outcomes [26,27,28], and previous studies with GBMSM in Kenya have identified high levels of social support as protective [11,29,30,35]. Our findings are consistent with previous research and are also consistent with the concept of social integration, which finds that having a strong social network and multiple sources of social support facilitates positive mental health [27,36]. A key finding from this study is the centrality of sexuality-specific social support across all sources and types of support discussed by Kenyan GBMSM.

Sexual orientation identity acceptance was identified as a form of emotional support in all relationship types that provided emotional support. With other GBMSM—friends and peer groups, sexual and romantic partners, and peer educators—participants received not only acceptance but also support related to their sexual orientation identity, with listening and sharing similar experiences as GBMSM highlighted as an important manifestation of emotional support by these groups. Though many participants did not identify their family of origin as a source of emotional support, those who did receive sexuality-specific emotional support emphasized the large positive impact this had on their well-being. Regarding healthcare providers, sexual orientation acceptance enabled providers to treat GBMSM with respect and dignity, allowed GBMSM to be honest about their health-related needs, and created an environment in which providers could truly listen to their GBMSM patients. Within workplaces, sexual orientation acceptance from colleagues allowed GBMSM to be open about their sexual orientation and feel “free”, which was perceived by participants to positively influence health.

Knowledge and acceptance of GBMSM’s sexual orientation was also vital for the provision of informational and instrumental support across sources of support. Knowledge and acceptance of men’s sexual orientation was required for informational support to be relevant and impactful, e.g., for healthcare providers to share appropriate sexual-health promotion strategies and for friends and peer groups to share information about GBMSM-friendly organizations. In this study, participants described informational support from their family of origin and healthcare providers as focusing specifically on health-related information, and informational support from sexual and romantic partners as focusing on strategies and information related to sexual-health promotion, while informational support from friends and peer groups and peer educators broadened beyond health-related information to also include information on raising awareness about LGBTQ identities and issues and sharing information about local GBMSM-friendly organizations and resources. Similarly, knowledge and acceptance of men’s sexual orientation was required for instrumental support to be relevant and impactful, e.g., for friends and peer groups to share strategies for coping with sexual minority stigma and for many groups to facilitate connections with GBMSM-friendly clinics and organizations. Though police officers were not described as providing emotional support, their ability to provide instrumental support in the form of protection of GBMSM from discrimination and victimization—as opposed to perpetrating victimization against GBMSM—relies on their understanding and acceptance of men’s sexual minority orientation.

Although we identified themes by source and type of social support, participants’ overall experience of support interacted across sources and types of support. Strong network connections facilitated men’s awareness of LGBTQ-focused organizations and resources and GBMSM-friendly clinics. Supportive families of origin facilitated identity pride and provided financial support so that men could finish their education, while strong connections to friends and peer groups and sexual and romantic partners buffered experiences of non-supportive family members by providing emotional and financial support. Throughout discussions, the strong influence of advocacy in the community was noted. For example, though police officers are often described as perpetrators of stigma and violence against GBMSM in Kenya, both historically and presently, several participants also described supportive experiences with police officers “who understand”, which could be related to recent efforts by local LGBTQ organizations to provide sensitization programs to police on the human rights of LGBTQ populations.

### 4.1. Study Implications

The current study identifies different types of social support from different groups of people that promote the health and well-being of GBMSM in Kenya. The types of support that individual GBMSM received interacted across their social networks, demonstrating the positive impact of participants’ social integration on their health and well-being [26,27,36]. In particular, our findings highlight the importance of sexuality-specific support as foundational to the provision of impactful social support to GBMSM and add to the extant literature by identifying potential resilience-promoting processes occurring at the interpersonal level. For example, based on our findings that social support from friends, peers, and romantic and sexual partners promotes both mental and physical health, health promotion programs for GBMSM in Kenya could find ways to integrate the involvement of GBMSM friends and peers, as well as romantic and sexual partners. For example, programs could provide opportunities to bring a partner to attend counseling sessions together, or program participants could bring friends to group sessions focused on health and well-being, to learn about ways to encourage and support one another in the goals they set (while stressing the importance of privacy and limits to confidentiality). Additionally, resources and programs could be developed to share information about how to access and utilize social support from friends, peers, and/or partners, as well as information about how to assess what is helpful and what is harmful in these relationships.

Peer educators were found to play an important role in facilitating GBMSM’s access to healthcare by bridging the gap between community members and GBMSM-friendly clinics [37]. This was consistent with previous studies showing the influence of peers and the necessity of engaging GBMSM in the development and delivery of services to improve health equity for this population [38]. The shared identities between peer educators and GBMSM clients allow for greater trust, more mutual understanding, and more open communication than many GBMSM have with healthcare providers. We found that peer educators also play a pivotal role in GBMSM’s awareness and acceptance of their own sexuality through one-on-one discussions, hosting forums to discuss issues affecting local GBMSM, and connecting individuals to local LGBTQI organizations and community resources. These connections made by peer educators facilitated individuals’ friendships and networking with other GBMSM, from whom they received additional social support, as well as engagement in community-level advocacy efforts, leading to individuals often becoming peer educators themselves. Our findings indicate that peer-delivered health promotion programs are acceptable and desired by GBMSM communities in Kenya, and that provision of social support by peer educators has the potential to create a snowball effect of resilience-promoting relationships and opportunities for GBMSM. Programs and clinics may amplify this impact by providing continued professional development training for peer educators, including topics related to mental health and information on how to appropriately provide all three types of social support (emotional, informational, and instrumental) to their clients. Additionally, resources could be developed to help GBMSM community members learn how to communicate their needs to peer educators, such as information about healthy and unhealthy relationship dynamics, effective communication skills, and information about the variety of ways that peer educators are able to provide support to community members (e.g., by arranging a ride to the clinic, delivering medication, or lending a listening ear), as well as information about what it is inappropriate for peer educators to do (e.g., lending money or providing psychotherapy).

This study demonstrated the centrality of sexuality-specific support to the provision of beneficial emotional, informational, and instrumental support by groups outside the LGBTQ community, including families of origin, healthcare providers, and others including work colleagues and police. Most research has focused on Kenyan families’ responses to learning that their child has a minority sexual orientation and has identified stigmatizing and rejecting behaviors including being kicked out of the family home, deprived of tuition payments and other previously provided economic support, and physical violence [6,39]. While this is not inconsistent with our research, our findings demonstrate variability in the response of Kenyan families; some families of origin are accepting of GBMSM family members or, even if not accepting of their sexual orientation, still provide love and support. Support may slowly increase over time or initially be limited to one member of the family of origin and then eventually expand to additional members of the family [40]. To assist families of origin to understand, accept, and support their LGBTQ family members, culturally grounded programs that provide information and resources on sexual orientation and how to support LGBTQ family members are recommended.

While many participants experienced stigma and discrimination in healthcare settings, especially at public facilities and clinics that were not LGBTQ-specific, other participants described the positive influence of supportive and accepting healthcare providers on their health and well-being, including their willingness to attend healthcare appointments. This is consistent with previous research indicating the impact of healthcare providers’ attitudes on ART adherence among GBMSM living with HIV [9]. Training programs that sensitize healthcare providers to the health-related needs of GBMSM have been shown to improve the care provided to GBMSM [41], and based on our findings, we recommend that these training efforts be expanded. The need for healthcare providers who are both knowledgeable and accepting of LGBTQ persons extends beyond sexual healthcare, especially to mental health-focused programs, counseling services, and substance-use treatment [20]. In recent years the Kenya Ministry of Health has prioritized a focus on mental health [42], and more local LGBTQ organizations are hiring or want to hire mental health counselors but can rarely identify mental health professionals who are accepting and knowledgeable about the lives of LGBTQ people. Community members note the need for mental health services from someone with whom they can talk about everything, who will understand their sexuality and be able to take a holistic approach. Additionally, increasing the use of client-centered approaches to GBMSM across healthcare disciplines may amplify the positive impact of support from healthcare providers. Research on PrEP uptake and adherence among GBMSM in Kenya indicates that even in GBMSM-friendly clinics, a large percentage of GBMSM who agree to take PrEP are not sufficiently adherent to reach protective levels of the drug and still hold stigmatizing or false conceptions of the medication [43]. The use of client-centered approaches such as Next Step Counseling [44,45,46] that build upon GBMSM’s current sources of social support and allow them to explore their own thoughts, motivations, and concerns regarding using PrEP (or other health promotion behaviors) may be more effective in health promotion and risk reduction counseling with GBMSM than traditional directive and top-down approaches.

Lastly, outside these relationship categories, participants also described receiving emotional support from work colleagues as affirmation of their sexual orientation, and some police officers provided instrumental support by protecting community members from discrimination and violence. As with some of the other relationship categories, the experience of receiving social support from work colleagues or police officers was by no means ubiquitous. Many GBMSM experience intense employment discrimination and/or are unable to be open about their sexual orientation identity at work [6]. However, our findings demonstrate that some GBMSM are open about their sexual orientation and receive identity-affirming support from their colleagues. Some of these identity-affirming workplaces include local GBMSM-friendly clinics and LGBTQ organizations focused on health and human rights. Many GBMSM in Western Kenya work or volunteer with LGBTQ-affirming clinics and organizations as community health workers and peer educators, simultaneously contributing to the overall health and social connectedness of their communities [21]. This work should be recognized and valued by local communities, and clinics and organizations serving GBMSM should prioritize hiring GBMSM community members in salaried positions. Similarly, interactions with police officers are often not positive. Although sexual activity between men is criminalized, it is not illegal to identify as LGBTQ or GBMSM, and the human rights of LGBTQ people are protected by the Kenyan Constitution [1,2,3]. However, due to pervasive societal stigma, there are many reports of police ignoring the victimization of GBMSM, or actually being the perpetrators of discrimination and violence against GBMSM, extorting money from GBMSM under threat of arrest and exposure [6]. Given this, it is encouraging that some GBMSM in our study had received help when needed from police. Over the past few years, a local grassroots LGBTQ organization developed and conducted training with area police departments, using a human rights perspective to sensitize police to the needs and experiences of local LGBTQ communities [47]. More “homegrown” approaches to reducing societal stigma against LGBTQ populations and training programs developed in partnership with LGBTQ Kenyans are recommended, not only for police and places of employment but also for families of origin, healthcare providers, and others who often have a large impact on an individual’s life and well-being but were not described by the GBMSM in this sample as providing social support, such as teachers and religious leaders [4,6,8].

### 4.2. Future Research

This study provides an initial exploration of the sources and types of social support that GBMSM in Western Kenyan receive. Future research should seek to understand additional sources of beneficial social support. In addition, future research should identify situations and contexts where social support is health-promoting for GBMSM, and identify situations and contexts in which various types of social support may actually be damaging for GBMSM (e.g., potential situations such as informational support that provides stigmatizing information about gay men or instrumental support that breaks confidentiality). Additionally, future research should work to develop programming that builds on the current findings by co-developing programs with GBMSM to be led by GBMSM that include a focus on building networks of social support, as well as training and sensitization programs that reduce stigma and increase acceptance of LGBTQ people among families, healthcare providers, police, religious leaders, and policymakers.

### 4.3. Stengths and Limitations

A strength of this study included our use of qualitative and participatory methodologies and use of a phenomenological approach to analysis, which allowed us to center community voices and gain a nuanced understanding of how GBMSM in Kenya experience social support. This represents a needed paradigm shift away from more commonly used deficit-based models and toward strengths-based approaches that highlight the resilience and resistance that communities of gay and bisexual men in Kenya consistently demonstrate in the face of pervasive oppression and marginalization. The overall experience of the research team was also a strength. Senior members of the research team have worked closely with GBMSM and the broader LGBTQ community in Kisumu for more than 10 years, including through community-engaged research, programming, clinical care, and advocacy. Study interviewers, who were trusted leaders of local grassroots organizations serving GBMSM, helped to develop and refine the interview guides, conducted the interviews, and helped to interpret the results. Multiple levels of triangulation during data collection (e.g., data collection by five different local interviewers, collecting data from two groups of key informants) and analysis (e.g., working with six analysts with varying educational levels and disciplinary backgrounds) was also a strength.

There were also limitations to the current study. The sexual-health focus of the overarching interview means that sexual health-related content was emphasized over other areas. Sources and types of social support were not included in the study’s primary research question, so participants were asked about these in different ways throughout the interview. This probably limited the identification of additional people who provide social support to GBMSM, within these interviews. For example, within the broad category of “other people”, participants only identified emotional support from work colleagues and instrumental support from police officers. However, from informal discussions and personal life experiences, we know that some GBMSM in Western Kenya receive emotional, informational, and instrumental support from additional sources such as religious leaders who provide LGBTQ-affirming services at a local community-based organization. Therefore, the current study should not be considered an exhaustive exploration of sources of social support. Additionally, the initial analysis was conducted only by U.S.-based researchers, though Kenyan members of the research team participated in a series of member-checking meetings and discussions about how to interpret the findings. Finally, these results may not be generalizable to all GBMSM in Kenya, though our sample of 60 participants is large for a qualitative sample and included viewpoints from GBMSM community members as well as GBMSM with significant experience of working as peer health educators within the community.

## 5. Conclusions

GBMSM receive social support from many different types of relationships, including friends and peer groups, families of origin, sexual and romantic partners, healthcare providers, peer educators, and other people including work colleagues and police officers. An important finding was the centrality of sexuality-specific support such as sexual orientation acceptance in enabling the provision of other types of support. Other GBMSM who were friends and peers, partners, and peer educators were instrumental to self-acceptance, coping with stigma, building social networks, and connecting to information and resources. Future health promotion programs can capitalize on these findings by employing GBMSM to implement peer-led programs that include a focus on building social support networks. It is imperative that such efforts include meaningful participation of local GBMSM, including opportunities for paid employment and professional development opportunities for GBMSM serving as peer health educators, community health workers, and research staff. Among non-LGBTQ groups, sexual orientation acceptance also facilitated their provision of relevant emotional, informational, and instrumental support to GBMSM. The fact that some GBMSM received sexuality-specific social support from sources that often stigmatize and reject them indicates that investing in sensitization programs and training to decrease stigma and increase acceptance of LGBTQ Kenyans among families, healthcare providers, police, religious leaders, and policymakers will improve the health of GBMSM.

## Figures and Tables

**Table 1:** Sample Demographics.

	Community Members (*n* = 40)	Peer Educators (*n* = 20)	Combined (*n* = 60)
Age			
	Mean = 26.4 years (range: 20–30)	Mean = 26.6 years (range: 22–45)	Mean = 26.4 years (range: 20–45)
Sexual Orientation			
Gay	16 (40.0%)	7 (35.0%)	23 (38.3%)
Bisexual	19 (47.5%)	7 (35.0%)	26 (43.3%)
MSM	5 (12.5%)	4 (20.0%)	9 (15.0%)
Other (wrote in: transgender *)	0 (0%)	2 (10.0%)	2 (3.3%)
Highest Educational Level			
Primary School	1 (2.5%)	1 (5.0%)	2 (3.3%)
Secondary School	11 (27.5%)	7 (35.0%)	18 (30.0%)
Certificate	6 (15.0%)	5 (25.0%)	11 (18.3%)
Diploma	15 (37.5%)	5 (25.0%)	20 (33.3%)
Bachelor’s Degree	4 (10.0%)	0 (0%)	4 (6.7%)
Master’s Degree	0 (0%)	1 (5.0%)	1 (1.7%)
Currently attending school	3 (7.5%)	1 (5.0%)	4 (6.7%)
Current Employment			
Part-time	16 (40.0%)	15 (75.0%)	31 (51.7%)
Full-time	4 (10.0%)	1 (5.0%)	5 (8.3%)
Casual Laborer	5 (12.5%)	0 (0%)	5 (8.3%)
Sex Worker	2 (5.0%)	2 (10.0%)	4 (6.7%)
Not working/in school	3 (7.5%)	0 (0%)	3 (5.0%)
Not working/not in school	4 (10.0%)	1 (5.0%)	5 (8.3%)
Other	6 (15.0%)	1 (5.0%)	7 (11.7%)
Religion			
Christian	37 (92.5%)	17 (85.0%)	54 (90.0%)
Muslim	3 (7.5%)	3 (15.0%)	6 (10.0%)
Ethnic Tribe			
Luo	35 (87.5%)	16 (80.0%)	51 (85.0%)
Luhya	3 (7.5%)	1 (5.0%)	4 (6.7%)
Digo	1 (2.5%)	1 (5.0%)	2 (3.3%)
Baganda	0 (0%)	1 (5.0%)	1 (1.7%)
Other	1 (2.5%)	1 (5.0%)	2 (3.3%)
Length of time as Peer Educator			
Less than 1 year	N/A	1 (5.0%)	N/A
Between 1 and 2 years	N/A	11 (55.0%)	N/A
Between 2 and 5 years	N/A	6 (30.0%)	N/A
More than 5 years	N/A	2 (10.0%)	N/A

All values are presented as total of a sample (*n*) and percent of representation (%) within those groups. * In the demographic survey, two participants selected “other” as their sexual orientation and wrote in “transgender”; both selected “male” as their assigned sex at birth and “male” as their current gender identity, so were considered eligible for the study and are included in the sample.

**Table 2:** Summary of types of social support provided by interpersonal relationship type.

	Emotional Support	Informational Support	Instrumental Support
	Things people do to make one feel loved, cared for, and worthy.	Help provided through the provision of information.	Things people do to provide tangible help.
Relationship Type			
Friends and Peer Groups	Acceptance and understanding through shared experiences as GBMSM; listen and share similar experiences as GBMSM; have fun together	Share information about health, well-being, and GBMSM-friendly organizations	Connect with GBMSM community; share coping strategies; provide medication reminders
Family of Origin	Sexual orientation identity acceptance; listen to feelings	Advise on how to maintain good health	Support to obtain education; provide medication reminders
Sexual and Romantic Partners	Sexual orientation identity support and acceptance; listen and share similar experiences as GBMSM; provide love and care; support health-related decisions	Share strategies and information related to sexual health protection	Financial support; connect with LGBTQ organizations and GBMSM-friendly clinics; engage in sexual risk reduction practices; provide medication reminders
Healthcare Providers	Sexual orientation identity acceptance; treat with respect and dignity; listen to needs	Share credible health information, including sexual-health promotion strategies; assist with informed decision-making	Provide medical services; work with peer educators to meet patients where they are; provide medication reminders
Peer Educators	Sexual orientation identity support and acceptance; listen and share similar experiences as GBMSM	Raise awareness of LGBTQI identities and issues; share information about health and local resources	Connect with LGBTQ organizations and GBMSM-friendly clinics; make medical appointments; deliver medication; provide transportation to clinic
Work Colleagues	Sexual orientation identity acceptance		
Police			Protection from discrimination and victimization

## Data Availability

The data presented in this study may be available on request from the corresponding author. The data are not publicly available due to the sensitive and potentially identifying nature of the qualitative data collected.
